# Supplementary material for: Effect of Drought Stress on Degradation and Remodeling of Membrane Lipids in Nostoc flagelliforme
Source: Foods. 2022 Jun 18;11(12):1798. doi: 10.3390/foods11121798 (PMC9222375; doi:10.3390/foods11121798)
Supplement: Supplementary file 1 [file foods-11-01798-s001.zip › foods-1748471 Supplementary figures R2.pdf]

foods

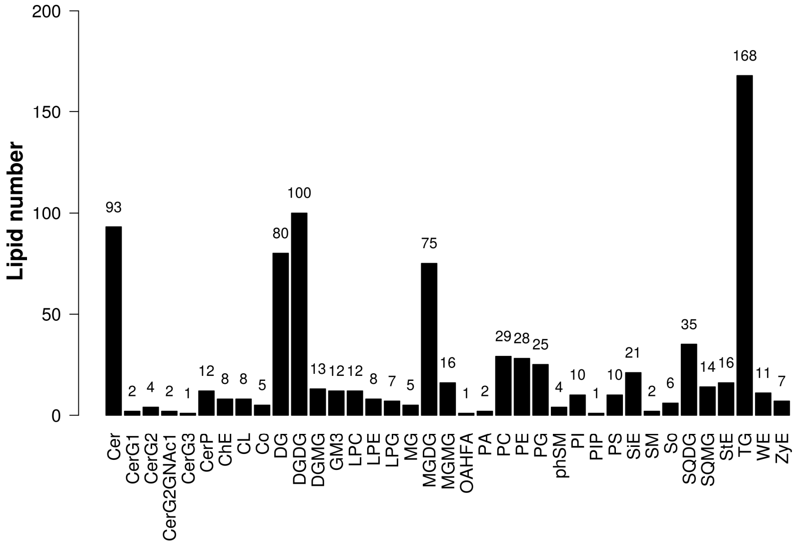

Figure S1. The number of lipid classes and lipid species in *N. flagelliforme* during drought stress. The abscissa in the figure represents the detected lipid classes and the ordinate is the number of lipid species.

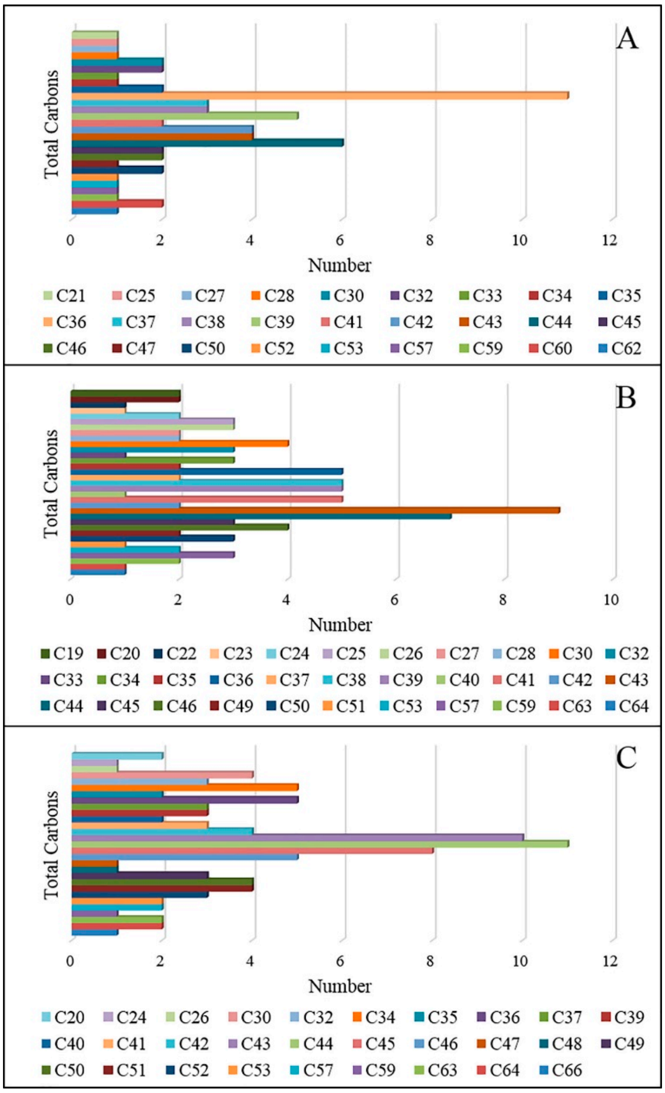

Figure S2. The total carbon chain length of DELs identified from *N. flagelliforme* under drought stress. A. Total carbon chain length of significant difference lipid species in LB group compare with LA group. B. Total carbon chain length of significant difference lipid species in LC group compare with LA group. C. Total carbon chain length of significant difference lipid species in LD group compare with LA group. LA, LB, LC, and LD represent samples with 0%, 30%, 75%, and 100% water loss, respectively.

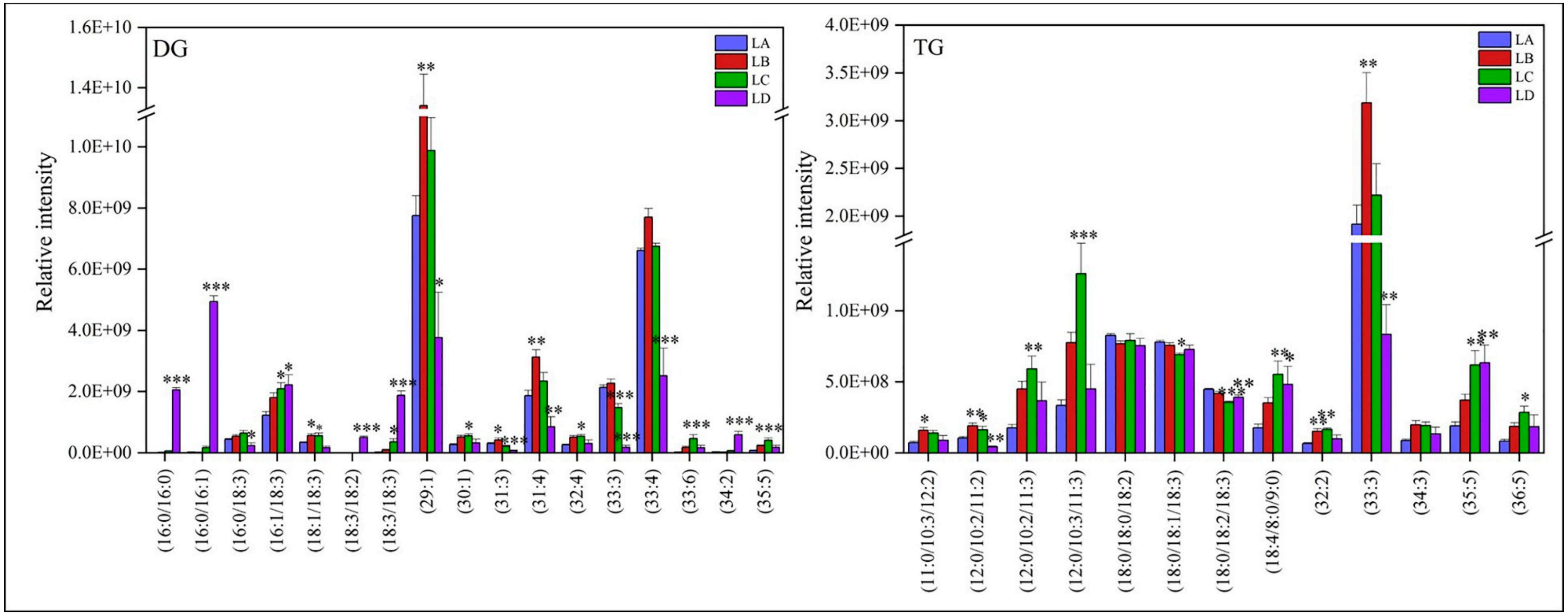

Figure S3. The changes of lipid molecular species of glycerolipid in *N. flagelliforme* under drought stress. LA, LB, LC, and LD represent samples with 0%, 30%, 75%, and 100% water loss, respectively. Values were presented as the means ± SE of eight independent biological samples. According to Student's t-test (*$p < 0.05$, **$p < 0.01$, and ***$p < 0.001$), asterisks above the bars indicate significant differences between the treatment groups (LB, LC, and LD) and the control group (LA).

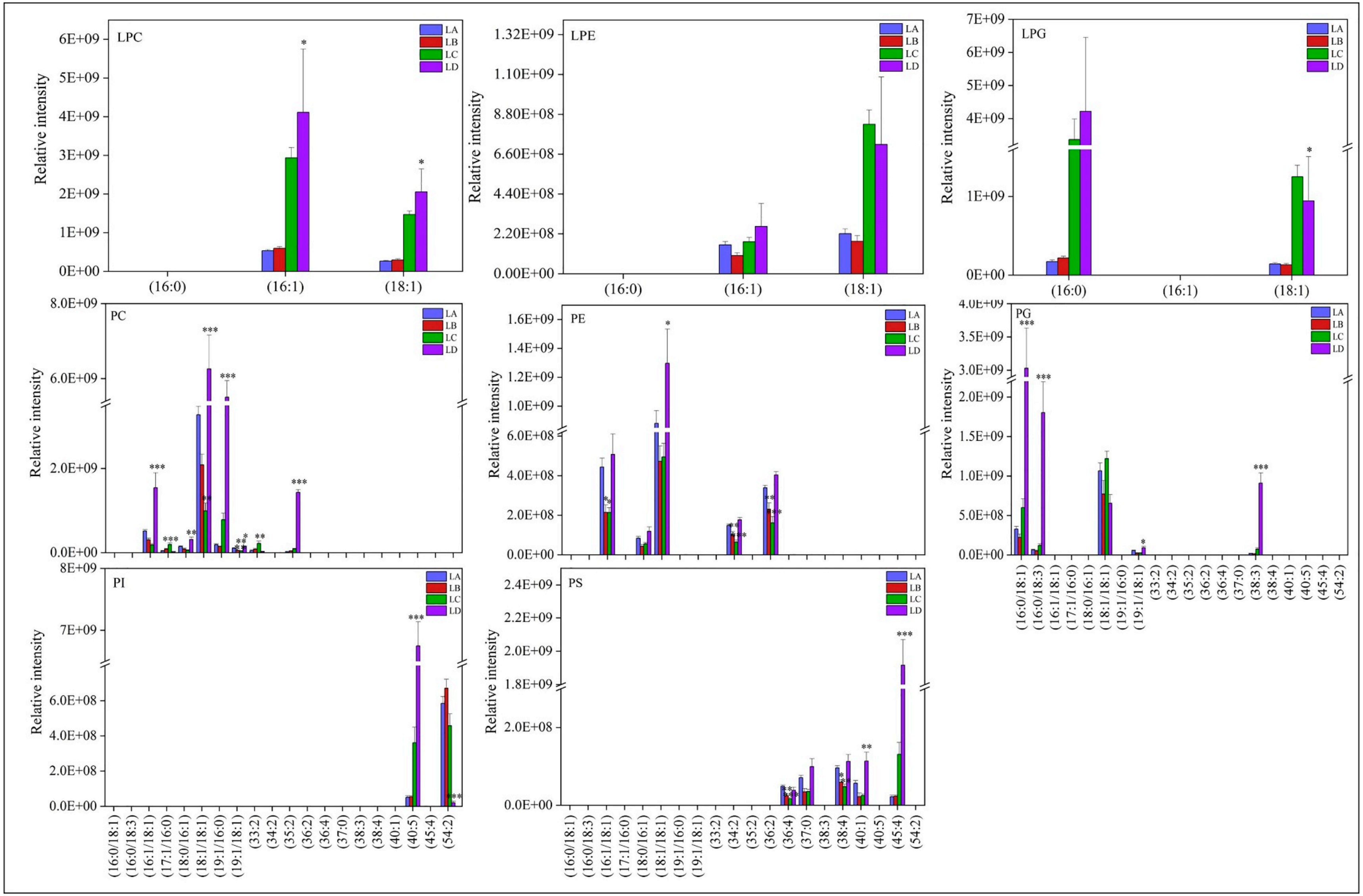
LPC
LPE
LPG
PC
PE
PG
PI
PS
LA
LB
LC
LD
Relative intensity
(16:0)
(16:1)
(18:1)
(16:0/18:1)
(16:0/18:3)
(16:1/18:1)
(17:1/16:0)
(18:0/16:1)
(18:1/18:1)
(19:1/16:0)
(19:1/18:1)
(33:2)
(34:2)
(35:2)
(36:2)
(36:4)
(37:0)
(38:3)
(38:4)
(40:1)
(40:5)
(45:4)
(54:2)

Figure S4. The changes of lipid molecular species of glycerolphospholipid in *N. flagelliforme* under drought stress. LA, LB, LC, and LD represent samples with 0%, 30%, 75%, and 100% water loss, respectively. Values were presented as the means ± SE of eight independent biological samples. According to Student's t-test (*p < 0.05, **p < 0.01, and ***p < 0.001), asterisks above the bars indicate significant differences between the treatment groups (LB, LC, and LD) and the control group (LA).

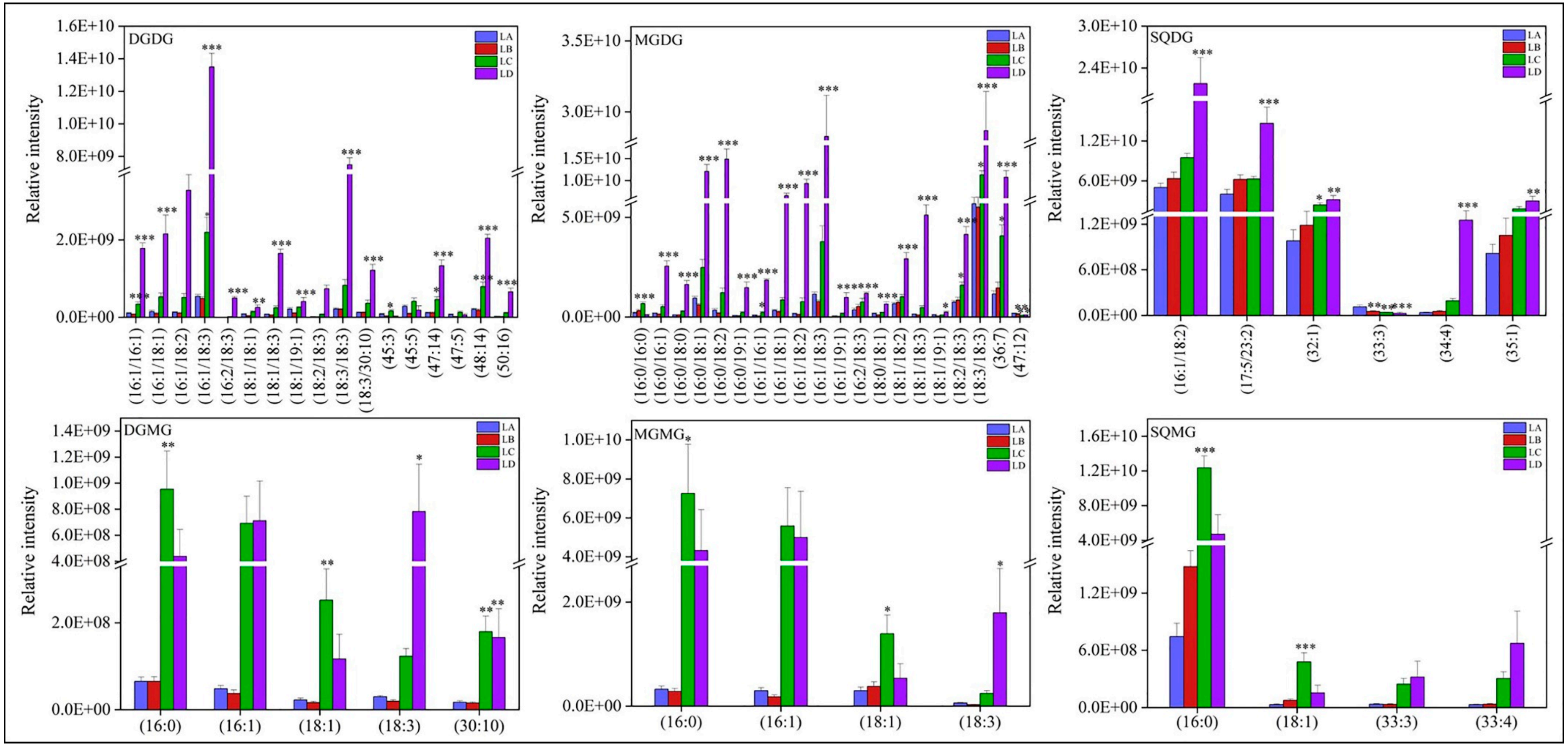

Figure S5. The changes of lipid molecular species of saccharolipid in *N. flagelliforme* under drought stress. LA, LB, LC, and LD represent samples with 0%, 30%, 75%, and 100% water loss, respectively. Values were presented as the means ± SE of eight independent biological samples. According to Student's t-test (*p < 0.05, **p < 0.01, and ***p < 0.001), asterisks above the bars indicate significant differences between the treatment groups (LB, LC, and LD) and the control group (LA).

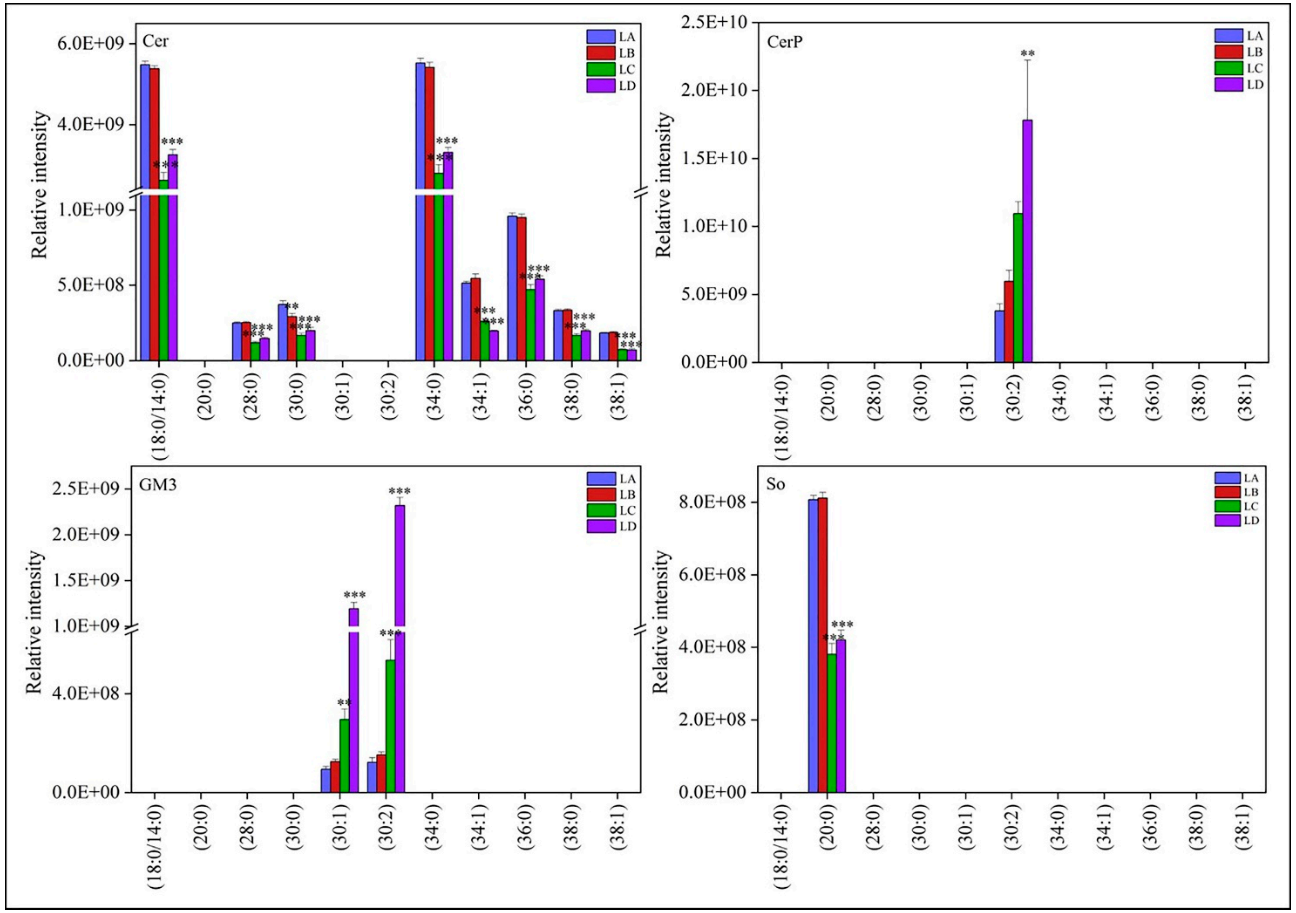

Figure S6. The changes of lipid molecular species of sphingolipid in *N. flagelliforme* under drought stress. LA, LB, LC, and LD represent samples with 0%, 30%, 75%, and 100% water loss, respectively. Values were presented as the means ± SE of eight independent biological samples. According to Student's t-test (*$p < 0.05$, **$p < 0.01$, and ***$p < 0.001$), asterisks above the bars indicate significant differences between the treatment groups (LB, LC, and LD) and the control group (LA).

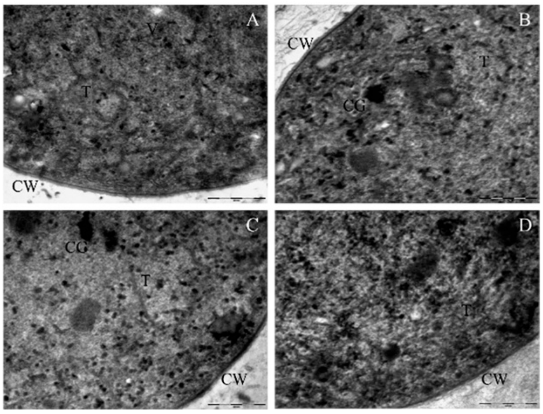

Figure S7. Ultrastructure of thylakoid in vegetative cell of *N. flagelliforme* subjected to dehydration. A: water loss 0 % (control); B: water loss 30 %; C: water loss 75 %; and D: water loss 100 % treatments. T: thylakoids, CG: cyanophycin granules, CW: cell wall, V: vacuoles. Scale bars equal to 200 nm.

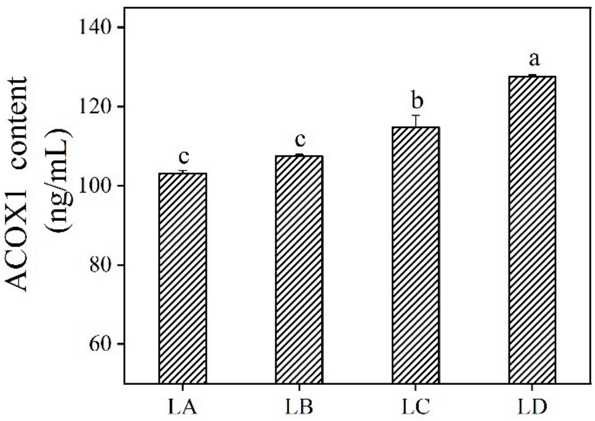

Figure S8. The content of ACOX1 related to lipid metabolism in *N. flagelliforme* under drought stress. Values were presented as the means ± SE (n = 3). The different letters above the bars indicate the means were significantly different ($p < 0.05$). LA, LB, LC, and LD represent samples with 0%, 30%, 75%, and 100% water loss, respectively.
